# Tightly Coupled LiDAR-Inertial Odometry and Mapping for Underground Environments

**DOI:** 10.3390/s23156834

**Published:** 2023-07-31

**Authors:** Jianhong Chen, Hongwei Wang, Shan Yang

**Affiliations:** School of Resources and Safety Engineering, Central South University, Changsha 410083, China

**Keywords:** LiDAR-Inertial odometry, underground environments, NanoGICP, IMU pre-integration

## Abstract

The demand for autonomous exploration and mapping of underground environments has significantly increased in recent years. However, accurately localizing and mapping robots in subterranean settings presents notable challenges. This paper presents a tightly coupled LiDAR-Inertial odometry system that combines the NanoGICP point cloud registration method with IMU pre-integration using incremental smoothing and mapping. Specifically, a point cloud affected by dust particles is first filtered out and separated into ground and non-ground point clouds (for ground vehicles). To maintain accuracy in environments with spatial variations, an adaptive voxel filter is employed, which reduces computation time while preserving accuracy. The estimated motion derived from IMU pre-integration is utilized to correct point cloud distortion and provide an initial estimation for LiDAR odometry. Subsequently, a scan-to-map point cloud registration is executed using NanoGICP to obtain a more refined pose estimation. The resulting LiDAR odometry is then employed to estimate the bias of the IMU. We comprehensively evaluated our system on established subterranean datasets. These datasets were collected by two separate teams using different platforms during the DARPA Subterranean (SubT) Challenge. The experimental results demonstrate that our system achieved performance enhancements as high as 50–60% in terms of root mean square error (RMSE).

## 1. Introduction

Humans have increasingly explored underground environments in the past two decades, leading to a growing demand for autonomous exploration and mapping of various subterranean settings, including tunnels, urban underground areas, and caves [1]. However, it is excessively risky for personnel to enter such environments due to significant variations, degradation, or temporal changes in hazards across domains. Thus, the development of underground simultaneous localization and mapping (SLAM), which enables navigation in underground environments with high-precision real-time localization and mapping without prior maps and the Global Position System (GPS), has become increasingly important.

Robust SLAM systems enable the extraction of humans from hazardous situations, such as search, rescue, and disaster response, while also enhancing efficiency in applications, such as surveying and automated mining in subterranean environments. Although the SLAM field has seen significant open-source developments in recent years, these systems primarily focus on open aboveground environments. In complex underground environments, there remain many challenges that affect the stable operation of SLAM systems.

One primary problem is that robots in underground environments typically lack the source of absolute positioning, such as surface vehicles (e.g., GNSS, RTK), due to the inability of GPS signals to penetrate the earth [2]. Another crucial problem is lighting. In many typical underground environments or disaster scenes, there is a lack of proper and homogeneous illumination, making it challenging to deploy visual and visual-inertial SLAM solutions that depend on distinctive visual textures [3]. Studies [4] have shown that even when the robot carries onboard light and creates illumination throughout the exploration, it is more difficult to utilize the visual information since the irregularities of the ground cause the movement of the light. The experiment in [4] reveals that even with the use of excellent ORB-SLAM2 [5], obtaining meaningful position estimation results in the underground environment is difficult.

Beyond cameras, another mainstream of SLAM is based on LiDAR sensors, which obtain tens of thousands of stable depth measurement points of surrounding environments every second and are not affected by poor lighting. The combination of LiDAR and IMU can obtain a robust estimation of a vehicle’s position in open-ground environments. However, it is flawed for the potential presence of dense obscurants, such as fog, swirling dust clouds, and smoke in underground environments. In environments such as long corridors and large open spaces, LiDAR odometry based on feature matching or point cloud registration is prone to failure due to the lack of perceptual features. Moreover, the complex and ambiguous terrain topography is further exacerbated by moving from a large cave to a small tunnel. In such environments, sudden changes in scale can cause a SLAM system which relies on a specific type of filter (e.g., voxel filter) to reduce computation time to failure if the parameter is fixed, but the environment has changed dramatically.

In this paper, we propose a tightly coupled LiDAR-Inertial odometry that focuses on location and mapping in underground environments. The main contributions of this paper are as follows:

(1) We adopted a point cloud segmentation method based on a range image to remove unstable points caused by airborne dust and the segmentation of ground points (for ground vehicles).
(2) To address the challenges of transitioning between different spatial conditions, we proposed an adaptive voxel filter. This filter maintains a fixed number of filtered points per frame, preventing loss of accuracy when moving from wider to narrower spaces and avoiding increased computational time and memory usage when moving from narrower to wider spaces.
(3) We proposed an indirect tightly coupled LiDAR-Inertial odometry system based on NanoGICP and IMU pre-integration via incremental smoothing and mapping. Indirect coupling facilitates robust and efficient estimation of the vehicle’s position.
(4) We extensively evaluated our proposed system compared with various open-source systems on datasets collected from representative underground environments.

The remainder of this paper is organized as follows: In Section 2, we discuss relevant research on underground SLAM. Section 3 provides an overview of the LiDAR-Inertial odometry framework and presents a detailed system overview. In Section 4, we present the results of a range of experiments conducted on datasets collected by teams participating in the Final Event of the DARPA SubT Challenge. Finally, we conclude in Section 5.

## 2. Related Work

This section presents a concise overview of SLAM research conducted in underground environments, focusing on notable solutions proposed in recent years.

The exploration of SLAM applications in subterranean environments has a significant history, with early works dating back to 2003. Thrun et al. [6] and Nuchter et al. were among the pioneers in conducting research in this domain. Their approach involved utilizing either a human-powered cart or a remote-control robot equipped with a laser range finder to generate volumetric maps of subterranean environments. Another noteworthy contribution was made by Tardioli et al. [7], who employed a team of robots to explore underground tunnels. Zlot et al. [8] developed a comprehensive 3D SLAM solution utilizing a spinning 2D lidar and an industrial-grade MEMS IMU. This system was capable of continuously acquiring data and successfully mapping a dense and accurate georeferenced 3D surface model over a distance of 17 km in under two hours. The advancements in SLAM research in underground environments have paved the way for further exploration and development in this challenging domain.

In recent years, numerous exceptional open-source SLAM solutions utilizing LiDAR as the primary sensor have emerged. These schemes can be classified into three categories: pure LiDAR odometry, loosely coupled odometry, and tightly coupled odometry, based on sensor type and optimization methods.

In 2014, Zhang et al. introduced LiDAR odometry and mapping (LOAM) [9]. LOAM is recognized as one of the most influential LiDAR-inertial-based works, pioneering feature-based LiDAR odometry. By minimizing the distance between corresponding edge and planar point features extracted in successive sweeps, LOAM iteratively estimates ego-motion until convergence occurs at a frequency of 10 Hz. Subsequently, features from the front end of each sweep are matched with the generated map to estimate the sweep’s position in the map frame at a lower frequency of 1 Hz. Building upon LOAM, Qin Tong released A-LOAM [10] as an open-source project. A-LOAM employs the nonlinear optimization library Ceres, developed by Google, to calculate residuals and enhance its readability. Wang et al. proposed the computationally efficient F-LOAM [11], which is based on LOAM and operates at a frequency of 20 Hz. F-LOAM computes the LiDAR distortion through frame-to-frame matching and subsequently updates it during the frame-to-map matching step once the current frame’s position is estimated. Guo et al. proposed E-LOAM [12] as a solution for unstructured environments. To address the issue of sparse features in such environments, this system incorporates a point cloud around feature points to provide additional local structure information. Furthermore, it utilizes a point cloud with sharp intensity changes as feature points. These additional sources of information enable the system to obtain more accurate estimation results.

Loosely coupled schemes refer to the operation of LiDAR and IMU as two independent subsystems within a LiDAR-Inertial system. The data from each sensor is typically processed and estimated independently using separate algorithms and methods. Subsequently, data fusion techniques are employed to combine the measurements from both sensors, resulting in more accurate and robust localization and motion estimation. Shan et al. introduced LeGO-LOAM [13] as an extension of LOAM. This system assumes flat ground for ground vehicles and extracts ground plane features as well as stable non-ground edge features from each scan. It employs a two-step optimization approach to solve for the different components of the position using these two types of features. Team CoSTAR released a loosely coupled LiDAR-Inertial odometry solution [14] known as direct LiDAR odometry. Direct LiDAR odometry utilizes a customized point cloud alignment method that combines NanoFlann [15] and FastGICP [16] to minimize the computational time required for point cloud alignment and covariance calculation. This approach achieves remarkable localization and mapping results even on computationally limited robotic platforms.

In contrast to the previously mentioned loosely coupled schemes, the tightly coupled scheme combines the original observations of LiDAR and IMU for joint processing, taking into account their intrinsic relationship and mutual influence, while fully considering the inherent constraints between these observations. The tightly coupled scheme can be categorized into two main approaches: optimization and filter.

He et al. introduced an open-sourced LIO-Mapping [17]. By minimizing the cost factor from LiDAR and IMU measurements, this system demonstrates good performance even in challenging environments where the quality of LiDAR measurements may be degraded. Shan et al. proposed LIO-SAM [18], which is based on the incremental smoothing and mapping framework iSAM2 [19]. This system heavily relies on feature matching to provide a LiDAR odometry factor that constrains the bias of a pre-integrated IMU factor exclusively. Furthermore, LIO-SAM requires a 9-axis IMU’s orientation as a supplementary input for position estimation, which may not be accurate in underground environments where magnetic fields can exist. Li et al. proposed LiLi-OM [20], which is suitable for both solid-state and mechanical LiDARs. This system utilizes a feature-based LiDAR odometry as the front end. The back end consists of a keyframe-based sliding window optimization, which directly fuses IMU and LiDAR measurements.

Regarding filter-based orientation, the first open-sourced system, LINS, in this domain was proposed by Qin et al. [21]. This system also assumes flat ground and employs an iterated error-state Kalman filter (ESKF) to recursively correct the estimated state, ensuring the computational tractability of the system. Successively, Xu et al. introduced FAST-LIO [22] and FAST-LIO2 [23]. The former incorporates a precise consideration of the LiDAR points’ sampling time through a formal backpropagation technique. Additionally, this system utilizes a new Kalman gain formula to reduce computational complexity. However, the system is only applicable to small environments due to the increasing time required for building a k-d tree of the updated map. The latter scheme, FAST-LIO2, utilizes a novel data structure called ikd-Tree, supporting incremental map updates and efficient k-nearest neighbors (KNN) inquiries. In a similar vein, Bai et al. [24] proposed Faster-LIO, which builds upon FAST-LIO2 and adopts incremental voxels as the point cloud spatial data structure instead of ikd-Tree. Leveraging this new data structure, Faster-LIO achieves a processing rate exceeding 200 Hz for 32-line spinning LiDAR while maintaining the same level of accuracy.

## 3. Materials and Methods

We begin by establishing the frames and notations that will be utilized throughout this paper. We use a set of points $p\in R^{3}$ with Cartesian coordinates composing a point cloud $P_{j}$ of the scan of timestamp ***j***. $S_{j}$ and $G_{j}$ refer to the noise-free point cloud and ground point cloud, respectively, corresponding to this scan. Additionally, $M_{j}$ represents the submap associated with this scan. For rotation representation, we use both R∈SO(3) and quaternions q. The world frame is denoted as W, while the robot body frame is denoted as B. The transformation matrix $T^{BW}\in SE(3)$ represents the transformation from B to W and can be represented as $T=\left[R|P\right]$. The robot state ***X*** is expressed as follows:

(1) $$X={\left[R^{T},p^{T},v^{T},b_{a}^{T},b_{g}^{T}\right]}^{T}$$

where $p\in R^{3},v\in R^{3}$ are, respectively, the position and linear velocity vector. $b_{a}$ and $b_{g}$ are the IMU accelerometer and gyroscope biases.

**Figure 1:**
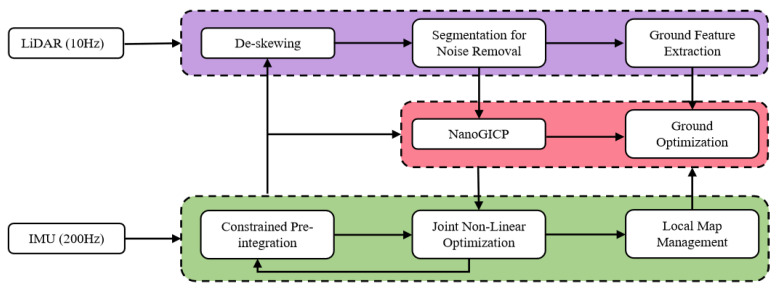
System pipeline.

A brief overview of our system’s pipeline is shown in Figure 1. Since the LiDAR suffers from the problem of motion distortion in one scan, once a scan of the point cloud is obtained, we first pre-integrate the IMU measurements between the time interval of two neighbor scans to obtain a relative motion approximation. This approximate relative motion estimation allows us to perform a de-skewing of the point cloud. Subsequently, with knowing the ring, azimuth, and range of each point in the current scan, we can project the de-skewed point cloud onto a range image. Each valid point is represented by a pixel in the range image, with the pixel value indicating the Euclidean distance from the origin. To address potential issues caused by air contaminants, such as dust and water mist, we employ a segmentation method proposed in [25] to filter out unstable points. If the system is employed on a ground vehicle, the ground point cloud is filtered simultaneously. In the next step, the filtered and stable point cloud is registered with a neighboring local map, constructed from selected keyframes. This registration process is performed using the robust and efficient NanoGICP algorithm. To reduce the time required for point cloud registration, we calculate an initial estimation of the relative transformation matrix between the current and previous scans using raw IMU readings while accounting for constrained IMU bias. The bias is determined based on the previous loop, and a new bias is obtained by combining the current transformation matrix from point cloud registration and the raw IMU readings for the prediction of the next loop. In our system, we choose the position and rotation change thresholds of 0.1 m and 30 degrees, respectively, as criteria for adding a new keyframe.

Our work attempts to address the following problem: leveraging continuously acquired low-frequency LiDAR point cloud measurements and high-frequency IMU measurements in underground environments to estimate the vehicle’s position and gather the environment information. To model this problem, we employ a factor graph that incorporates IMU pre-integration factors and LiDAR factors for graph construction through incremental smoothing and mapping. The optimal state of the keyframe, denoted as x, is obtained by minimizing the following expression:

(2) $$\underset{x}{\min}\left\{{\left\|r_{i}\right\|}_{\Sigma_{i}}^{2}+{\left\|r_{l}\right\|}_{\Xi_{i}}^{2}\right\},$$

where $r_{i}$ is the residual of IMU pre-integration and $r_{l}$ defines the residual of relative LiDAR constraint, respectively. $\Sigma_{i}$ and $\Xi_{i}$ represent their associated covariances.

### 3.1. Adapt Voxel Grid Filter

Using a voxel filter is an efficient way to preserve the environmental structure while reducing the number of points in a scan, particularly when there is excessive redundancy. This situation often arises when a robot transitions between narrow aisles and open environments, or vice versa. Additionally, it is common to encounter scenarios involving multiple LiDAR configurations on a vehicle, resulting in an overwhelming number of points that cannot be processed in real time. When employing a fixed-size voxel filter in such situations, it becomes challenging to find suitable voxel values capable of accommodating all scenarios.

To address the computational burden of LiDAR odometry and ensure a consistent computation time per scan, irrespective of environmental changes and LiDAR configurations, we propose an adaptive voxel grid filter. This easy but efficient approach allows us to maintain a roughly constant number of voxelated points (approximately 10,000 points in our system) instead of specifying a fixed voxel leaf value. By doing so, we mitigate the impact of varying input points originating from different sensor configurations and environments characterized by significant spatial variations.

The approach is as follows: Initially, we select an initial voxel leaf size, denoted as $d_{init}$, and set $d_{leaf}=d_{init}$. Here, $d_{leaf}$ represents the size of the voxel leaf in the current scan. We introduce $N_{curr}$ to represent the number of points after applying voxel filtering, and $N_{desire}$ to represent the desired number of points. To control the voxel grid size effectively, we propose the following control equation:

(3) $$d_{{leaf}_{t+1}}=\left\{\begin{array}{c} 0.98\times d_{{leaf}_{t}},if\frac{N_{curr}}{N_{desire}}<0.95\\ 1.02\times d_{{leaf}_{t}},if\frac{N_{curr}}{N_{desire}}>1.05 \end{array}\right.,$$

The formula defines the voxel leaf size for the next scan, denoted as $d_{{leaf}_{t+1}}$, by considering the ratio between the number of points in the current filtered scan $N_{curr}$ and the desired number of points for computation $N_{desire}$ given the robot’s configuration. This straightforward technique enables us to maintain a consistent number of points, preventing significant fluctuations that may result in either an insufficient number of points (e.g., in narrower environments with a relatively large voxel size) or an excessive number of points. As a result, the system achieves enhanced efficiency and reduced computational burden.

### 3.2. Segmentation of Noise and Ground Points

#### 3.2.1. Segmentation for Noise Removal

Most underground environments exhibit challenging conditions, characterized by the presence of swirling dust clouds and so on. These factors pose significant obstacles to the reliable use of LiDARs in such environments. Therefore, it becomes crucial to address and mitigate these adverse effects as they introduce uncertainties in point cloud registration. Fortunately, by employing a simple configuration or calculation, we can readily obtain the ring and azimuth values for each point captured by a mechanical LiDAR and project the raw point cloud onto a range image by leveraging this information.

To group the pixels effectively, we adopt the segmentation method proposed in [25]. Specifically, for each point in the current point cloud, excluding the first and last rows of the range image, we examine the four neighboring points located above, below, to the left, and to the right of the current point. It is important to note that the points situated on the left and right boundaries, as well as the opposite side of the boundary, also form adjacent pairs due to the horizontal scanning process and physical continuity. If the connecting line between two neighboring points in either the horizontal or vertical direction is approximately perpendicular to the current beam, we consider these points to belong to the same object. By applying a threshold value (approximately 30 in our system) to the cluster of points belonging to an object, we can identify stable clusters. Any cluster with a number of points below this threshold is deemed unstable and subsequently filtered out.

#### 3.2.2. Segmentation for Ground Points

The limited vertical resolution and vertical field of view of LiDAR data per scan result in challenges for ground detection, leading to drift in the z-axis for pure LiDAR odometry. To address this, many systems incorporate an IMU as an additional sensor to create LiDAR-Inertial odometry, which provides better constraints on roll and pitch angles. However, cumulative error and long-term z-axis offset still pose issues for mapping. A valid solution is to extract the ground point cloud by leveraging ground plane features and creating residuals with the ground plane cloud of the local map. Ground optimization, although dependent on the flat ground assumption, has proven effective in existing systems. In our system, we also extract the ground point cloud by utilizing the projected range image, especially in the case of using a ground vehicle.

We primarily focus on the lower half of the range map since it contains point clouds predominantly directed toward the ground. To determine whether a point belongs to the ground, we calculate two metrics using Equations (4) and (5) for two points, $p=\left(x_{p},y_{p},z_{p}\right),\;q=(x_{q},y_{q},z_{q})$, located in the same column and adjacent row of the range image.

(4) $$\theta =atan2(z_{p}-z_{q},\sqrt{{\left(x_{p}-x_{q}\right)}^{2}+{\left(y_{p}-y_{q}\right)}^{2}})$$

(5) $$d=\sqrt{{\left(x_{p}-x_{q}\right)}^{2}+{\left(y_{p}-y_{q}\right)}^{2}+{\left(z_{p}-z_{q}\right)}^{2}}$$

We will treat these two points as ground points if the condition that |θ|<10 and d<1 is met.

### 3.3. Tightly Coupled LiDAR-Inertial Odometry

#### 3.3.1. IMU Pre-Integration Factor

The measurements of angular velocity and acceleration from an IMU are defined using (6 and 7).

(6) $${\hat{\omega }}_{t}=\omega_{t}+b_{t}^{\omega }+n_{t}^{\omega },$$

(7) $${\hat{a}}_{t}=R_{t}^{BW}{(a}_{t}-g)+b_{t}^{a}+n_{t}^{a},$$

where ${\hat{\omega }}_{t}$ and ${\hat{a}}_{t}$ denote the raw IMU measurements in IMU coordinate at time t. The $b_{t}$ denotes the bias that will additively affect the ${\hat{\omega }}_{t}$ and ${\hat{a}}_{t}$ slowly. The $n_{t}$ denotes the white noise. The $R_{t}^{BW}$ is the rotation matrix from W to B, and g denotes the constant gravity vector in W.

According to the IMU pre-integration method proposed by [26], since IMU measurements are sampled at a very high rate, we can assume a constant angular velocity and acceleration for the time of integration between two IMU measurements. All measures between two frames or keyframes at times k=i and k=j can be summarized in a single compound measurement, which constrains the motion between two moments using (8–10).

(8) $$R_{j}=R_{i}\prod_{k=i}^{j-1}Exp(({\hat{\omega }}_{k}-b_{k}^{\omega }-n_{k}^{\omega d})\Delta t),$$

(9) $$v_{j}=v_{i}+g{\Delta t}_{ij}+\sum_{k=i}^{j-1}R_{k}^{WB}({\hat{a}}_{k}-b_{k}^{a}-n_{k}^{ad})\Delta t,$$

(10) $$p_{j}=p_{i}+\sum_{k=i}^{j-1}\left[v_{k}\Delta t+\frac{1}{2}g{\Delta t}^{2}+\frac{1}{2}R_{k}^{WB}({\hat{a}}_{k}-b_{k}^{a}-n_{k}^{ad}){\Delta t}^{2}\right],$$

where ${\Delta t}_{ij}=\sum_{k=i}^{j-1}\Delta t$ and ${\left(∙\right)}_{i}\dot{=}(∙)(t_{i})$. The Exp(∙) denotes the exponential map of a vector, as suggested in [27], $Exp:\;R^{3}\to SO(3)$. Δt denotes the sampling period of the IMU. The $n^{\omega d}$ denotes the discrete-time noise and the covariance of $n^{\omega d}$ is a function of the sampling rate and relates to continuous-time noise $n^{\omega }$ through $Cov(n^{\omega d}(t))=\frac{1}{\Delta t}Cov(n^{\omega }\left(t\right))$. The $n^{ad}$ has the same relations.

Whenever the linearization point changes at $t_{i}$, the Equations (8)–(10) need to be recomputed. To avoid this drawback, the following Equations (11)–(13) are used.

(11) $${\Delta R}_{ij}=\prod_{k=i}^{j-1}Exp(({\hat{\omega }}_{k}-b_{k}^{\omega }-n_{k}^{\omega })\Delta t),$$

(12) $$\Delta v_{ij}=R_{i}^{BW}\left(v_{j}-v_{i}-g{\Delta t}_{ij}\right)=\sum_{k=i}^{j-1}R_{ik}^{WB}\left({\hat{a}}_{k}-b_{k}^{a}-n_{k}^{ad}\right)\Delta t,$$

(13) $$\begin{array}{ll} \Delta p_{ij} & =R_{i}^{BW}\left(p_{j}-p_{i}-v_{i}{\Delta t}_{ij}-\frac{1}{2}g{{\Delta t}_{ij}}^{2}\right)\\ & =\sum_{k=i}^{j-1}\left[{\Delta v}_{ik}\Delta t+\frac{1}{2}{\Delta R}_{ik}^{WB}({\hat{a}}_{k}-b_{k}^{a}-n_{k}^{ad}){\Delta t}^{2}\right]. \end{array}$$

Thus, the residuals of the IMU pre-integration can be defined using the following equation.

(14) $$r_{i}=\left[\begin{array}{c} R_{i}^{BW}(p_{j}-p_{i}-v_{i}{\Delta t}_{ij}-\frac{1}{2}g{{\Delta t}_{ij}}^{2})-{\hat{\Delta p}}_{ij}\\ R_{i}^{BW}(v_{j}-v_{i}-g{\Delta t}_{ij})-{\hat{\Delta v}}_{ij}\\ \begin{array}{c} {2\left[q_{i}^{BW}⨂q_{j}^{WB}⨂{\hat{q}}_{ji}^{B}\right]}_{xyz}\\ \begin{array}{c} b_{j}^{a}-b_{i}^{a}\\ b_{j}^{\omega }-b_{i}^{\omega } \end{array} \end{array} \end{array}\right],$$

where ${\left[∙\right]}_{xyz}$ denotes the vector part of a quaternion q for the error-state representation. $\left[{\hat{\Delta p}}_{ij},{\hat{\Delta v}}_{ij},{\hat{q}}_{ji}^{B}\right]$ are pre-integrated IMU measurement terms, which are only affected by the bias of IMU and can be approximated by the first order approximation.

Building upon the forthcoming discussion on the LiDAR odometry factor in the next section, we can estimate a constrained bias. Utilizing this bias, we can then employ new IMU measurements to predict the robot’s motion and rectify the point cloud of the subsequent keyframe.

(15) $$R_{t+1}=R_{t}Exp\left(\left({\hat{\omega }}_{t}-b_{t}^{\omega }\right)\Delta t\right),$$

(16) $$v_{t+1}=v_{t}+g\Delta t+R_{t}^{WB}({\hat{a}}_{t}-b_{t}^{a})\Delta t$$

(17) $$p_{t+1}=p_{t}+v_{t}\Delta t+\frac{1}{2}g{\Delta t}^{2}+\frac{1}{2}R_{t}^{WB}\left({\hat{a}}_{t}-b_{t}^{a}\right){\Delta t}^{2}.$$

#### 3.3.2. LiDAR Odometry Factor

The LiDAR odometry uses the NanoGICP, which combines two models: NanoFLANN and FastGICP. The NanoFLANN can achieve fast nearest neighbor and less memory usage queries and the FastGICP achieves efficient point cloud registration through multithreaded parallel computing.

When a new frame $P_{k}^{c}$ arrives, we first calculate the position of the current point cloud using the estimated IMU bias in the last loop and the IMU samplings between the timestamp of the last frame of the point cloud and the current frame of the point cloud, utilizing (15–17). Then we build a submap $P_{k}^{m}$ of the current point cloud of no more than 10 historical keyframe point cloud. The objective is to compute a relative transform ${\hat{T}}_{k}^{WB}$ between the current point cloud $P_{k}^{c}$ and the submap point cloud $P_{k}^{m}$. The GICP algorithm [28] models the surface from which a point was sampled as a Gaussian distribution: $p_{i}^{c}\sim N{({\hat{p}}_{i}^{c},C}_{i,k}^{c}),\;p_{i}^{m}\sim N{({\hat{p}}_{i}^{m},C}_{i,k}^{m}),\;i=1IN$, for N pairs of corresponding points. The $C_{i,k}^{c}$ and $C_{i,k}^{m}$ are the corresponding estimated covariance matrices. Then the transformation error is defined as follows:

(18) $$d_{i}=p_{i}^{m}-T_{k}^{WB}p_{i}^{c},p_{i}^{c}\in P_{k}^{c},p_{i}^{m}\in P_{k}^{m}.$$

We can calculate the distribution of $d_{i}$ using the property of Gaussian distribution:

(19) $$d_{i}\sim N\left(p_{i}^{m}-T_{k}^{WB}p_{i}^{c},C_{i,k}^{m}+T_{k}^{WB}C_{i,k}^{c}T_{k}^{BW}\right)\sim N\left(0,C_{i,k}^{m}+T_{k}^{WB}C_{i,k}^{c}T_{k}^{BW}\right).$$

We want to find a transformation $T_{k}^{WB}$ that maximizes the log likelihood of (19).

(20) $${\hat{T}}_{k}^{WB}=\underset{T_{k}^{WB}}{argmax}\prod_{i}^{N}p(d_{i})=\underset{T_{k}^{WB}}{argmin}\sum_{i}^{N}log(p(d_{i})).$$

The (20) can be simplified to (21).

(21) $${\hat{T}}_{k}^{WB}=\underset{T_{k}^{WB}}{argmin}\sum_{i}^{N}d_{i}^{T}{\left(C_{i,k}^{m}+T_{k}^{WB}C_{i,k}^{c}T_{k}^{BW}\right)}^{-1}d_{i}.$$

The ground vehicle suffers from drift of the z-axis—optimizing for the ground can mitigate this drift. After obtaining the transformation matrix $T_{k}^{WB}$ through (21), it is still significant to utilize the ground feature to constrain the drift of roll, pitch, and z-axis of ground vehicles.

Following the approach described in [13], we extract features from the segmented ground points of the current scan, as discussed in Section 3.2.2. We use $r_{ij}$ to represent the depth of a ground point that is located in the ***i***-th row and ***j***-th column of the range image. Subsequently, we calculate the smoothness of the current point using the following equation.

(22) $$c=\frac{1}{10\times r_{ij}}\left\|\sum_{j-5\leq k\leq j+5,k\neq j}r_{ik}-r_{ij}\right\|.$$

**Figure 2:**
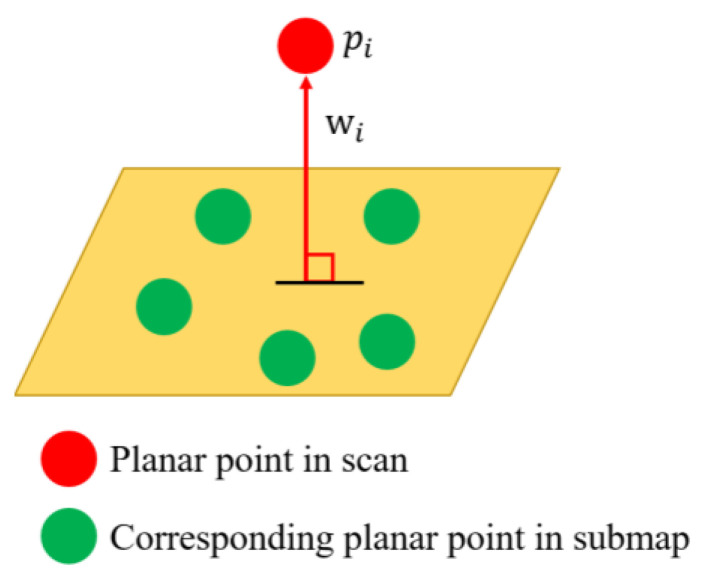
Planar residual.

A point is considered a ground feature if its curvature c is below a threshold (0.1 in our system). To extract the local ground features, we construct a kd-tree based on the ground local map and search for the five nearest points to the current ground feature point within the local ground map. By employing QR decomposition, we formulate a system of overdetermined equations for these five points, enabling us to compute the local plane parameters $[w,d],w\in R^{3}$. The plane residual for the ***i***-th plane feature point is computed using the following equation.

(23) $$r_{i}\left(p_{i},w_{i},d_{i},T^{WB}\right)=w_{i}^{T}\left(R^{WB}p_{i}+t^{WB}\right)+d_{i},$$

where $p_{i}$ is a plane feature point in the current scan and $\left[w_{i},d_{i}\right]$ is the corresponding plane parameter, as shown in Figure 2. For all ground features of the current scan, we minimize all residual errors to obtain the MLE using the following equation.

(24) $${\hat{T}}_{k}^{WB}=\underset{T_{k}^{WB}}{argmin}\sum_{i=1}^{N}r_{i}^{2}.$$

For ease of calculating the Jacobian matrix, we adopt Euler angle to represent the rotation matrix. Specifically, we use ***x y z*** to denote the translation of current scan B relative to W, and ***roll pitch yaw*** corresponding to the rotation angle of ***x y z*** axis of current scan B relative to W. So, the to be estimated parameter $T_{k}^{WB}$ can be represented as $x={\left[x,y,z,roll,pitch,yaw\right]}^{T}$. Then the rotation matrix can be represented as

(25) $$R^{WB}=\left[\begin{array}{ccc} cycp & cyspsr-crsy & sysr+cycrsp\\ cpsy & cycr+syspsr & crsysp-cysr\\ -sp & cpsr & cpcr \end{array}\right],$$

where cy denotes cos(yaw) and sy denotes sin(yaw), respectively.

Since we only estimate the ***r***, ***p*** and ***z*** of the current scan, the Jacobian of a plane feature point $p={\left[\begin{array}{ccc} p_{x} & p_{y} & p_{z} \end{array}\right]}^{T}$ is

(26) $$J_{i}={\left[\begin{array}{ccc} 0 & 0 & \begin{array}{ccc} \frac{\partial r_{i}}{\partial z} & \frac{\partial r_{i}}{\partial r} & \begin{array}{cc} \frac{\partial r_{i}}{\partial p} & 0 \end{array} \end{array} \end{array}\right]}^{T}$$

where

(27) $$\frac{\partial r_{i}}{\partial z}=w_{z},$$

(28) $$\begin{array}{ll} \frac{\partial r_{i}}{\partial r} & =w_{x}\left(\left(cyspcr+srsy\right)p_{y}+\left(sycr-cysrsp\right)p_{z}\right)\\ & +w_{y}\left(\left(-cysr+syspcr\right)p_{y}+\left(-srsysp-cycr\right)p_{z}\right)\\ & +w_{z}\left(cpcrp_{y}-cpsrp_{z}\right), \end{array}$$

(29) $$\begin{array}{ll} \frac{\partial r_{i}}{\partial p} & =w_{x}\left(\left(-cysp\right)p_{x}+\left(cycpsr\right)p_{y}+\left(cycrcp\right)p_{z}\right)\\ & +w_{y}\left(\left(-spsy\right)p_{x}+\left(sycpsr\right)p_{y}+\left(crsrcp\right)p_{z}\right)\\ & +w_{z}\left(\left(-cp\right)p_{x}+\left(-spsr\right)p_{y}+\left(-spcr\right)p_{z}\right), \end{array}$$

where ${\left[\begin{array}{ccc} w_{x} & w_{y} & w_{z} \end{array}\right]}^{T}=w$ denotes the plane parameter. At last, we can use Gauss–Newton to obtain the optimal to be estimated parameters.

(30) $$\sum_{i=1}^{N}J_{i}J_{i}^{T}\Delta x=-\sum_{i=1}^{N}J_{i}r_{i}.$$

Finally, we can obtain the transformation matrix $\Delta T_{k}^{k-1}$ between $X_{k-1}$ and $X_{k}$, which encodes the LiDAR odometry factor.

(31) $$\Delta T_{k}^{k-1}=T_{k-1}^{BW}T_{k}^{WB}.$$

## 4. Results and Discussion

We conducted an experimental evaluation of our algorithm on four datasets collected at the SubT Final Event held by DARPA in Louisville Mega Cavern. We compared our results with five state-of-the-art LiDAR or LiDAR-Inertial odometry methods: A-LOAM [10], Direct LiDAR Odometry (referred to DLO) [14], FAST-LIO2 [23], VoxelMap [29], and LiLi-OM [20]. However, we were unable to test LIO-SAM [18], despite it being one of the most influential and state-of-the-art tightly coupled approaches, due to its strict input data requirements.

The first two datasets were collected by Team CERBERUS, utilizing their legged robot based on the ANYmal C100 series quadruped by ANYbotics. The robot was equipped with a VLP16 LiDAR sensor and an Alphasense Core, a visual-inertial sensor by Sevensense [30]. Our system utilized only the LiDAR and IMU data. The remaining two datasets were collected by Team Explorer using an unmanned aerial vehicle (UAV) [31]. The UAV has VLP16 LiDAR and an IMU. We utilized only the LiDAR and IMU data. Table 1 provides a detailed description of the datasets. All the experiments were conducted on a computer with an Intel Core i5-11400 processor, 16 GB RAM, and Intel HD Graphics. The software platform used was Ubuntu 18.04 and the Robot Operating System (ROS) Melodic 18.04. Nonlinear optimization was performed using Ceres, while the factor graph optimization was solved using GTSAM [32].

### 4.1. Analysis of Segmentation

**Figure 3:**
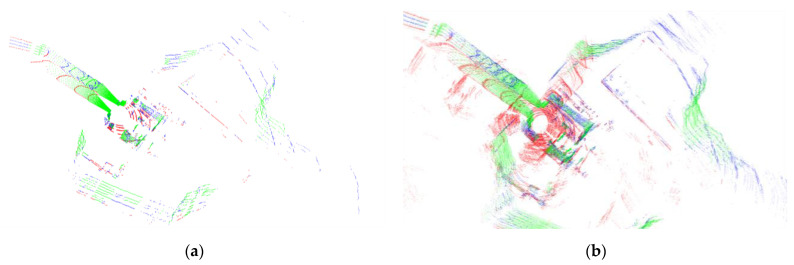
Segmented point cloud in sequence anymal1: red point cloud presents segmented ground, blue point cloud presents segmented unstable cloud. (**a**) A frame of point cloud after segmentation. (**b**) A submap of point cloud after segmentation.

Figure 3 presents the segmented point cloud of a ground vehicle from the anymal1 dataset. The ground point cloud is highlighted in red, while the unstable point cloud is marked in blue. It is evident that the ground point cloud and the unstable point cloud are distinctly separated.

### 4.2. Analysis of Adaptive Voxel Filter

**Figure 4:**
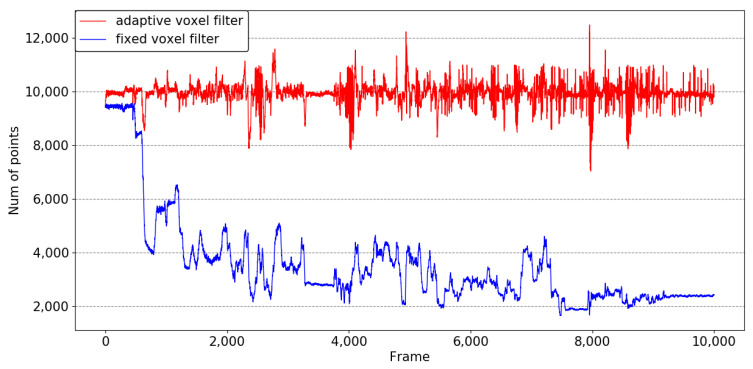
Results of adaptive voxel filter and fixed voxel filter.

We evaluate the performance of our adaptive voxel filter using the anymal1 sequence, where the vehicle transitions from relatively open areas to narrower lanes. The initial leaf value of the voxel filter is set to 0.1 m, and the desired number of points is 10,000. We select successive segments of 1000 s from the anymal1 sequence to calculate the number of points after applying different voxel filter. The results of this experiment are depicted in Figure 4. It is observed that the number of points after applying our adaptive voxel filter fluctuates around 10,000. In contrast, the fixed-sized voxel filter leads to a rapid decrease in the number of points per scan as the vehicle enters the roadway. The information loss caused by the fixed-sized voxel filter results in an increased error in position estimation. The experiment is introduced in Section 4.4.

### 4.3. Compared with Other Systems

We employed rpg-trajectory-evaluation [33] to assess the results in four sequences. However, for unknown reasons, LiLi-OM failed in all sequences; thus, we excluded this system from the table to conserve space. Additionally, Team CERBERUS included the results of their CompSLAM [34] for the two sequences they collected, which served as a benchmark for evaluating other researchers’ systems. We included the CompSLAM results in the table to compare the datasets they provided.

It should be noted that most open-sourced systems utilize fixed-sized voxel filters to reduce processing time, and they are primarily designed for open aboveground environments, such as streets, rather than narrow subterranean environments. Consequently, the voxel filter’s leaf size in these systems is relatively large, which is not suitable for testing in narrow subterranean environments. Additionally, the majority of systems employ geometric features (e.g., point-line features, point-polygon features) to construct cost functions for optimization. However, adopting the voxel filtering value recommended by the author would lead to the loss of numerous effective features, significantly compromising precision and accuracy. To ensure a fair comparison among systems, we opted for a relatively small leaf value for the voxel filter in the preprocessing stage of all systems. This decision was made to capture more detailed environmental information. Additionally, we provide the specific voxel filtering values for each system to enhance result reproducibility. Specifically, we employ a voxel filter with a leaf size of 0.05 m in the preprocessing step of A-LOAM, DLO, FAST-LIO2, and LiLi-OM. Notably, A-LOAM and LiLi-OM utilize both point-line and point-plane features and construct two independent feature submaps for feature matching. For the line submap, we set the voxel filter’s leaf size to 0.1 m, while for the plane submap, it is set to 0.2 m. Since all datasets were acquired using VLP16 LiDAR, we did not omit any line’s point cloud in any of the systems.

The estimation error between the ground truth $X_{gt}$ and the aligned estimation $X_{es}$ is evaluated using two widely adopted error metrics: the absolute trajectory error (ATE) and the relative error (RE). The ATE measures the global consistency of the trajectory and provides a straightforward numerical metric for comparing position estimates. The RE quantifies the local accuracy of the trajectory within a fixed time interval, reflecting its local consistency. For a more detailed understanding of the calculation metrics, we encourage readers to refer to [34].

Our evaluation primarily focuses on root mean square error (RMSE) of the four sequences, which provides better indications of the system’s robustness and stability. The results for absolute rotational drift (ARE), absolute translation drift (ATE), relative rotational drift (RRE), and relative translation drift (RTE) of four sequences are presented in Table 2, Table 3, Table 4 and Table 5, respectively.

We calculate the improvement value using the following equation.

(32) $$\eta =\frac{o-r}{o}\times 100\%,$$

where η represents improvement, o represents the result of other systems, and r represents the result of our system, respectively. We compared our system to the best-performing open-source systems in each sequence and demonstrated the improvements in RMSE and std as presented in Table 6. It should be noted that we did not compare CompSLAM as it incorporates various types of sensors. The improvements of our system can reach up to 50–60% in terms of RMSE across the four sequences.

**Figure 5:**
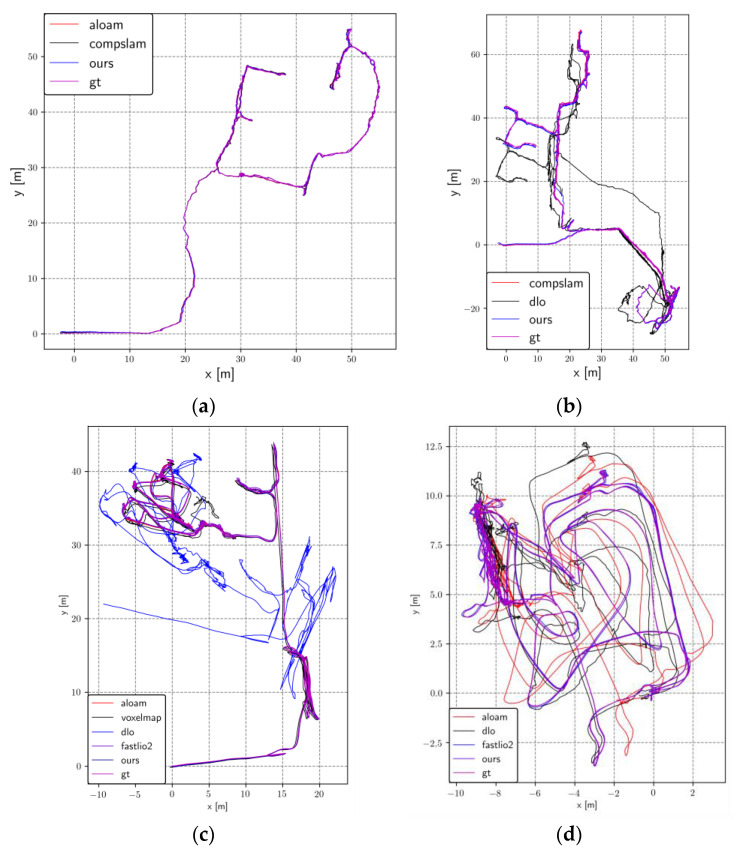
Comparison between the motion trajectories generated by different algorithms and ground truth. (**a**) Sequence anymal1; (**b**) Sequence anymal2; (**c**) Sequence ds3; (**d**) Sequence ds4.

**Figure 6:**
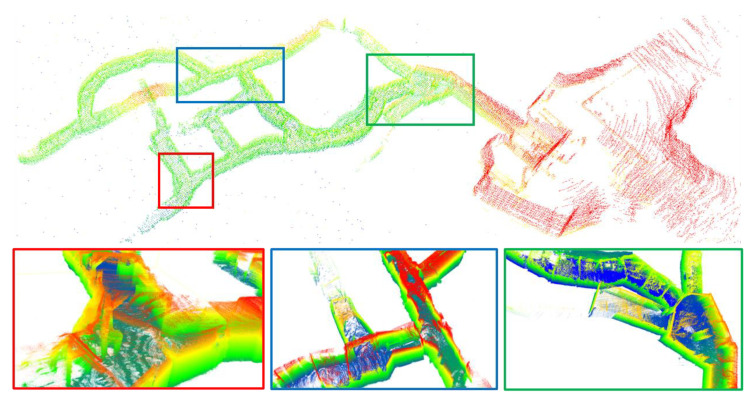
Different views of sequence anymal1 mapped by our system, colored with height.

**Figure 7:**
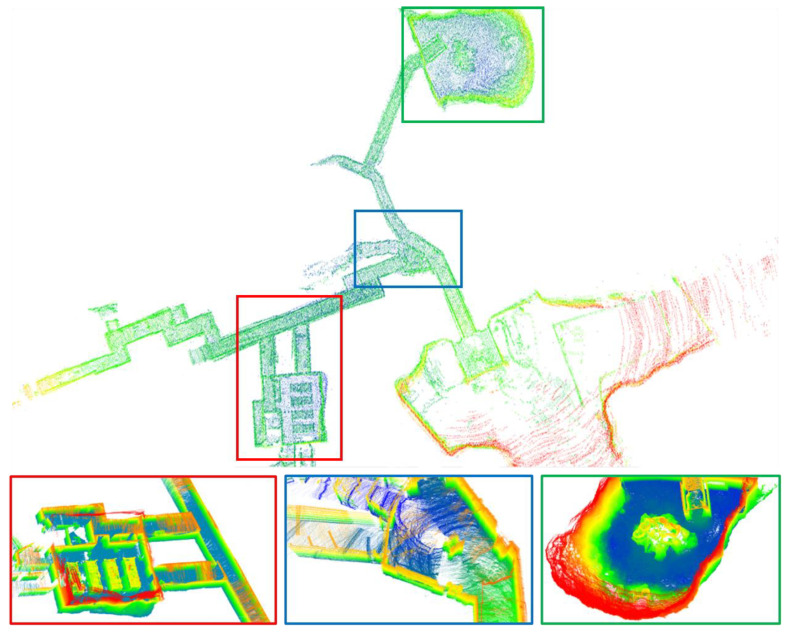
Different views of sequence anymal2 mapped by our system, colored with height.

Figure 5 illustrates the trajectory and ground truth mapping of all successfully evaluated systems on the x-y axis. It is evident that most systems evaluated on the two sequences collected by Team CERBERUS have failed. The CompSLAM system, proposed by Team CERBERUS and victorious in the Final Event of DARPA, has demonstrated successful alignment with the ground truth due to its loosely coupled multi-sensor fusion mechanism capable of handling diverse degradation and interference scenarios in the underground environment. In the remaining sequences, collected by Team Explorer using a UAV, many systems achieved successful mapping. However, as depicted in Figure 5d, although many systems exhibited trajectories similar to the ground truth, they were unable to achieve successful alignment with the ground truth. Our system delivered competitive results across all sequences. We present the mapping results of two sequences collected by Team CERBERUS using our system in Figure 6 and Figure 7.

**Figure 8:**
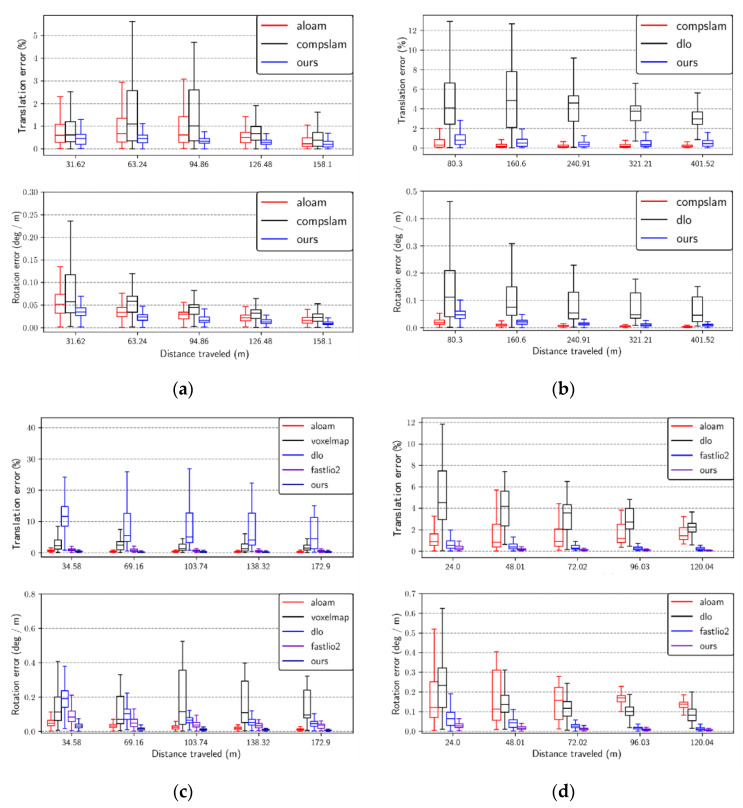
RE w.r.t. percentage of distance traveled from 10% to 50%. (**a**) Sequence anymal1; (**b**) Sequence anymal2; (**c**) Sequence ds3; (**d**) Sequence ds4.

Figure 8 displays the box plots depicting the relationship between RE and trajectory length for the translation and rotation components of each successfully mapped system. The x-axis represents the percentage of the traveled distance (ranging from 10% to 50%). The y-axis for the rotation component indicates the error per meter in degrees (deg/m), while the y-axis for the translation component represents the percentage of error. All results from the four sequences are presented in Figure 8 as a separate subplot from (a) to (d). On average, our system exhibits relatively smaller translation and rotation drift compared with most other systems. In Figure 8b, the result for sequence anymal2 is plotted. It is evident that the CompSLAM outperforms our system in both translational and rotational components. This difference in performance is likely attributed to the CompSLAM’s integration of diverse independent sources of odometry, such as visual, thermal, inertial, and kinematic, to ensure robustness in various perceptually degraded environments.

### 4.4. Ablation Experiment

In order to ascertain the essentiality of each component in our system for its proper functioning, we conducted a series of ablation experiments. We eliminated the point cloud segmentation component and the adaptive voxel filter component from our system, respectively, and compared the performance of the modified system with that of the complete system. It is important to note that the removal of the point cloud segmentation component results in the loss of ground optimization capability in our system. The experiment was conducted using the sequence anymal1, which exhibited significant spatial changes. The RMSE was computed for each subsystem, and the results are tabulated in Table 7.

**Figure 9:**
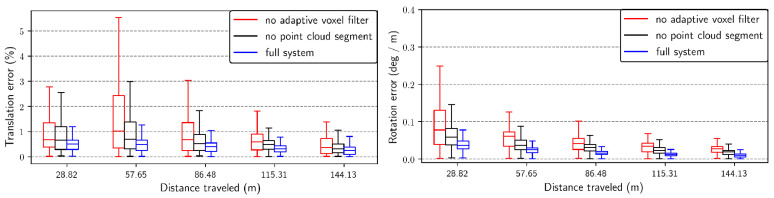
RE w.r.t. percentage of distance traveled from 10% to 50%.

Additionally, the relative error is plotted in Figure 9. The experiment demonstrates that disabling the adaptive voxel filter or cloud segmentation leads to a decline in system performance.

## 5. Conclusions

In this work, we present a tightly coupled online LiDAR-inertial odometry system, which accurately estimates the 6-DoF motion of the robot and efficiently maps underground environments. Our system benefits from the following features:

(1) Efficient segmentation of the ground point cloud and unstable point cloud using range image-based techniques to facilitate subsequent point cloud registration.
(2) An adaptive voxel filter that maintains the desired number of point clouds in each frame, preventing information loss due to fixed-size voxel filters in underground environments with significant spatial variations.
(3) An indirect tightly coupled LiDAR-Inertial odometry that employs NanoGI-CP and IMU pre-integration with factor graph optimization.

Using high-quality datasets collected by both teams in the DARPA SubT Finals using terrestrial and aerial platforms, we performed comprehensive evaluations of our system and various state-of-the-art open-source systems. The results demonstrate that our system surpasses many existing and state-of-the-art methods, particularly in challenging environments such as tunnels and corridors.

## Figures and Tables

**Table 1:** Details of all the sequences for the benchmark.

Dataset Name	Time(s)	Platform	LiDAR	IMU
anymal1	3750	Anymal-C	VLP-16	Alphasense 200 Hz
anymal2	3883	Anymal-C	VLP-16	Alphasense 200 Hz
ds3	619	UAV	VLP-16	Xsens 200 Hz
ds4	486	UAV	VLP-16	Xsens 200 Hz

**Table 2:** RMSE of Sequence anymal1.

Seq Anymal1	A-LOAM	CompSLAM	Ours
ARE (°)	1.8464	3.1072	1.1494
ATE (m)	0.2429	0.3458	0.1305
RRE (°/m)	0.0058	0.0059	0.0031
RTE (%)	0.1817	0.1989	0.0959

**Table 3:** RMSE of sequence anymal2.

Seq Anymal2	DLO	CompSLAM	Ours
ARE (°)	24.966	1.3477	2.8548
ATE (m)	9.4632	0.2045	0.7348
RRE (°/m)	0.0642	0.0019	0.0043
RTE (%)	2.4814	0.0799	0.4311

**Table 4:** RMSE of sequence ds3.

Seq ds3	A-LOAM	VoxelMap	DLO	FAST-LIO2	Ours
ARE (°)	2.057	16.95	24.23	5.580	1.332
ATE (m)	0.145	1.607	7.371	0.541	0.090
RRE (°/m)	0.005	0.156	0.023	0.013	0.004
RTE (%)	0.163	1.242	6.599	0.287	0.180

**Table 5:** RMSE of sequence ds4.

Seq ds4	A-LOAM	DLO	FAST-LIO2	Ours
ARE (°)	9.813	7.516	1.855	0.663
ATE (m)	1.151	1.511	0.208	0.055
RRE (°/m)	0.127	0.082	0.011	0.005
RTE (%)	2.240	1.795	0.207	0.053

**Table 6:** Improvement w.r.t best of other open-sourced systems.

Matrics	Anymal1	Anymal2	ds3	ds4	Average
ARE	37.75%	88.56%	57.12%	64.26%	61.92%
ATE	46.27%	92.23%	37.93%	72.39%	62.20%
RRE	46.55%	93.30%	20.00%	63.64%	55.87%
RTE	47.22%	82.63%	35.44%	79.59%	61.22%

**Table 7:** Result of ablation experiment.

Seq Anymal1	No Cloud Segmentation	No Adaptive Voxel Filter	Full System
ARE (°)	2.1627	1.5658	1.1494
ATE (m)	0.2502	0.2136	0.1305
RRE (°/m)	0.0436	0.0107	0.0031
RTE (%)	0.9606	0.3742	0.0959

## Data Availability

The data presented in this study are openly available at https://libdrive.ethz.ch/index.php/s/W626tMEBkROF8hG?path=%2FCERBERUS_Darpa_Subt_Final_Event (accessed on 14 February 2023) and https://superodometry.com/datasets (accessed on 17 January 2023).
